# Plant-parasitic nematodes associated with the root zone of hop cultivars planted in a Florida field soil

**DOI:** 10.21307/jofnem-2020-040

**Published:** 2020-04-23

**Authors:** Tristan T. Watson, Marco Suarez, Zhanao Deng, Johan A. Desaeger

**Affiliations:** 1 Department of Entomology and Nematology, Gulf Coast Research and Education Center, University of Florida, 14625 County Road 672, Wimauma, Florida, 33598; 2 Department of Plant Pathology and Crop Physiology, Louisiana State University, 302 Life Science Building, Baton Rouge, Louisiana, 70803; 3 Department of Environmental Horticulture, Gulf Coast Research and Education Center, University of Florida, 14625 County Road 672, Wimauma, Florida, 33598

**Keywords:** Hop cultivars, *Humulus lupulus*, *Meloidogyne javanica*, Nematode management, *Pratylenchus brachyurus*

## Abstract

In early 2016, hop plants were introduced into Florida. By late 2016, the hop plants were showing stunted growth and were heavily parasitized by *Meloidogyne javanica*. In this study, we determined host susceptibility of 14 hop cultivars to *M. javanica* in a greenhouse experiment and monitored population development of plant-parasitic nematode species in the root zone of 17 hop cultivars planted in three newly established hop yards in Florida. Plant-parasitic nematodes in the rooting zone soil of field grown hop plants included *M. javanica*, *Pratylenchus brachyurus*, *Paratrichodorus minor*, *Belonolaimus longicaudatus*, *Xiphinema setariae/vulgare* complex, *Mesocriconema xenoplax*, and *Helicotylenchus dihystera;* however, soil population densities of *P. minor*, *B. longicaudatus*, *X. setariae/vulgare* complex, *M. xenoplax*, and *H. dihystera* remained low through the study. Root galling, *M. javanica* egg production, and soil population densities of *M. javanica* were consistently large on the ‘Canadian Red Vine’, ‘Centennial’, ‘Chinook’, and ‘Comet’ cultivars, and small on the ‘Galena’ and ‘Triple Perle’ cultivars. No differences were observed in soil population densities of *P. brachyurus* among hop cultivars. Overall, our study provides the first report of plant-parasitic nematode population development in the root zone on hop cultivars planted in Florida.

Hop cones from the hop plant (*Humulus lupulus* L.) are a fundamental ingredient of the brewing process, imparting bitterness, aroma, and flavor to beer. In the United States, hop cultivation began when the first colonialists arrived in the 1600s (Turner et al., 2011). By the late 1800s, hop production spread to states in the Pacific Northwest (Steiner, 1973). Modern hop production is largely concentrated in Washington, Oregon, and Idaho, where favorable day length, climate, and low disease pressure allow for production of excellent quality cones and high yields; however, many other regions are now exploring hop as an alternative crop. The United States is the world’s leading hop producer, responsible for 41.3% of the crop produced worldwide (Hope Growers of America, 2018). Some of the most commonly planted hop cultivars include Citra^®^, Cascade, and Centennial (USDA-NASS 2018).

Although several bacterial, fungal, and viral diseases have been documented on hop (Mahaffee et al., 2009), there are relatively few reports on the plant-parasitic nematode species associated with this crop. The hop cyst nematode (*Heterodera humuli* Filipjev) is the most well studied nematode associated with hop worldwide (De Grisse and Gillard, 1963), and has been reported in United States hop production in Pierce County, Washington (Cobb, 1962). Other plant-parasitic nematodes associated with hop in the United States include *Meloidogyne hapla* Chitwood and *Xiphinema americanum* Cobb, which were found widely distributed within hop yards in Sacramento County, California (Maggenti, 1962), as well as *Pratylenchus neglectus* Filipjev & Schuurmans-Stekhoven and *Pratylenchus thornei* Sher & Allen, which have been reported less frequently within Idaho and Oregon (Hafez et al., 1992). In New Zealand, *Ditylenchus destructor* Thorne was isolated from severely stunted plants from commercial hop yards (Foot and Wood, 1982). Severe nematode damage in a hop yard in Belgium was associated with *H. humuli*, *Tylenchorhynchus dubius* Bütschli, *Pratylenchus penetrans* Filipjev & Shuurmans Stekhoven, and *Helicotylenchus* sp. (Pelerents et al., 1983). In China, *Meloidogyne incognita* Kofoid & White was reported on the hop cultivar Comet; however, nematode population development was restricted in a locally bred cultivar (Zhang and Zheng-Li, 1989).

As a result of the high demand for locally produced hop from the micro-brewing industry, hop plants were recently introduced into Florida as an alternative crop. In early 2016, the University of Florida planted hop rhizomes at the Gulf Coast Research and Education Center in Hillsborough County, Florida to evaluate the potential of this crop. By late 2016, some of the hop plants were showing stunted growth and yellow leaves. Uprooted plants exhibited severe root galling, and females extracted from galls, as well as J2s extracted from soil, were subsequently identified as *Meloidogyne javanica* (Brito et al., 2018).

In Florida, root-knot nematodes (*Meloidogyne* spp.) are widespread and likely pose a significant obstacle to any hop yard planted in the state. Many other plant-parasitic nematode species that are abundant in the region may also pose a threat to hop production. Using a combination of field and greenhouse experiments, the objectives of this study were to: (i) determine host susceptibility of 14 cultivars of hop to *M. javanica* in a greenhouse experiment, and (ii) monitor population development of plant-parasitic nematode species in the root zone of 17 cultivars of hop planted in three newly established hop yards in Florida.

## Materials and methods

### Site description

The studies were conducted at the Gulf Coast Research and Education Center research farm in Wimauma, Florida, United States. Soil at the site is an Arredondo fine sand (95% sand, 3% silt, 2% clay). The field had been fallow for over 10 years under natural grass cover prior to conducting the following trials.

### Hop yard planting No. 1

In 2016, a new hop yard was established at the research farm to evaluate plant-parasitic nematode population development on 10 hop cultivars from two planting material sources. Planting rows were 82-m long and were spaced 4.88-m apart, with a 0.91-m wide strip of black geotextile mulch covering the center of each row. Hills were spaced 0.91-m apart within the planting row. A 6.1-m tall trellis wire was installed above each row. Plants were irrigated with two drip tapes (30-cm emitter spacing), each located 0.46-m feet away from the row center along both sides of the planting row. Plants were fertilized with all-purpose fertilizer (20:20:20) at a rate of 149 kg N/ha/season. Ground cover between rows consisted of perennial ryegrass (*Lolium perenne*) that was mowed periodically throughout the growing season.

The experimental design was a randomized complete block with three blocks and 12 cultivar treatments. In total, 36 plots were overlaid on to three planting rows within the hop yard. Plots consisted of four hills (3.66-m long) and were planted at a density of two rhizomes per hill. A 0.91-m buffer zone was located between plots to minimize edge effects. On April 12, 2016, triplicate plots were planted with rhizomes from each of the following cultivars: (i) ‘CTZ’, (ii) ‘Cascade’ (source: Oregon), (iii) ‘Cascade’ (source: Washington), (iv) ‘Centennial’ (source: Oregon), (v) ‘Centennial’ (source: Washington), (vi) ‘Chinook’, (vii) ‘Fuggle’ (source: Oregon), (viii) ‘Magnum’ (source: Washington), (ix) ‘Nugget’ (source: Washington), (x) ‘Sorachi Ace’ (source: Washington), (xi) ‘Triple Perle’ (source: Washington), or (xii) ‘Willamette’ (source: Washington).

Plants were harvested in August 2016 and December 2016 by cutting the bines by hand at ground level. During the growing season, a number of the cultivars displayed symptoms of a viral disease, which was subsequently confirmed as *Apple Mosaic Virus* (ApMV). Due to the distribution and severity of the symptoms the hop yard was scheduled to be renovated using clean planting material in 2017.

In December 2016, plants were uprooted from the soil after harvest using a shovel and assessed for root galling. Entire root systems were assessed for galling and given a root gall index rating on a scale from 0 to 10 (Bridge and Page, 1980), with 0 indicating no visible galling and 10 indicating 100% galled. The abundance of plant-parasitic nematodes in soil was also determined for each plot. Eight soil cores (20 cm in length, 2.5 cm in diameter) were obtained from each plot directly from the previous rooting zone (two soil cores per hill). Soil samples were placed into plastic bags and were stored at 4°C for a maximum of 14 days prior to subsequent processing. Nematodes were extracted from a 200-mL subsample of soil from each plot using the Baermann pan technique (Forge and Kimpinski, 2007), with a two-day incubation period. After collecting the nematodes over a 25-μm sieve, the nematodes were transferred in water into plastic scintillation vials and store at 4°C for a maximum of 14 days prior counting on an inverted compound microscope.

### Hop yard planting No. 2

In 2017, a new hop planting was established in the previously renovated hop yard. On August 15, 2017, metam potassium (K-Pam^®^ HL; AMVAC Chemical Corporation, Los Angeles, CA) was drip applied to the previous planting rows at a rate of 584 L/ha. The experimental design was a randomized complete block with three blocks and 12 cultivar treatments overlaid on the previous plots from the first field experiment in 2016. Each hill within a plot was planted with two transplants. On September 13, 2017, triplicate plots were planted with tissue-cultured transplants from each of the following cultivars: (i) ‘Cascade’ (source: Washington), (ii) ‘Cascade’ (source: Florida), (iii) ‘Cashmere’, (iv) ‘Centennial’ (source: Washington), (v) ‘Centennial’ (source: Florida), (vi) ‘Chinook’ (source: Washington), (vii) ‘Chinook’ (source: Florida), (viii) ‘Comet’ (source: Washington), (ix) ‘Magnum’ (source: Washington), (x) ‘Nugget’ (source: Washington), (xi) ‘Triple Perle’ (source: Washington), or (xii) ‘Zeus’ (source: Washington).

In February 2018, artificial lights (Philips Flowering Lamp 2.0; Philips, Amsterdam, the Netherlands) were installed every 2.74 m along the trellis wire to extend the daylight length by 5 hr from March to May and from September to November. Plants were harvested by hand in December 2017, July 2018, December 2018, and July 2019 by cutting the bines at ground level.

The abundance of plant-parasitic nematodes in soil was determined on four sampling dates (December 2017, July 2018, December 2018, and July 2019). Soil was sampled and nematodes were quantified as described previously.

### Hop yard planting No. 3

In 2017, an additional new hop planting was established on a single planting row adjacent to the previous hop yard from 2016. On August 15, 2017, metam potassium was drip applied to the planting row, as described above. Planting rows were covered with a 0.91-m wide strip of metallic plastic mulch. The experimental design was a randomized complete block with four blocks and five cultivars overlaid on the previous plots from the first field experiment in 2016. Each hill within a plot was planted with two transplants. On September 15, 2017, plots were planted with tissue-cultured hop transplants of each of the following cultivars sourced from Washington: (i) ‘Canadian Red Vine’, (ii) ‘Galena’, (iii) ‘Mt. Rainier’, (iv) ‘Tahoma’, or (v) ‘Willamette’.

Artificial lighting was installed every 2.74 m along the trellis wire to extend the daylight length during March to May and September to November. Plants were harvested by hand in December 2017, July 2018, December 2018, and July 2019 by cutting the bines at ground level.

The abundance of plant-parasitic nematodes in soil was determined on three sampling dates (December 2017, July 2018, December 2018, and July 2019). Six soil cores were obtained from each plot directly from the previous planting holes (two cores per hill). Soil samples were processed, and nematodes were quantified as described previously.

### Greenhouse experiment

In 2017, a greenhouse experiment was performed to evaluate the susceptibility of 14 hop cultivars to *M. javanica*. The experimental design was a randomized complete block with six blocks and 14 cultivar treatments. In total, 84 20-cm diameter circular pots were filled with 3.5 L of soil mix (three parts steam sterilized field soil: two parts potting mix). The nematode inoculum was prepared by extracting eggs from a population of *M. javanica* maintained in a greenhouse on tomato roots using the sodium hypochlorite technique (Hussey and Barker, 1973), followed by washing the eggs in water over a 25-μm sieve. The eggs were inoculated as 1-mL of nematode egg suspension (2,500 eggs/mL) into each of four 1-cm deep holes made in the top of the soil in each pot (10,000 eggs/pot). After nematode inoculation, six replicate pots were planted on September 18, 2017 with a single tissue-cultured hop transplant from one of the following cultivars sourced from Washington: (i) ‘Canadian Red Vine’, (ii) ‘Cascade’, (iii) ‘Cashmere’, (iv) ‘Centennial’, (v) ‘Chinook’, (vi) ‘Comet’, (vii) ‘Galena’, (viii) ‘Magnum’, (ix) ‘Mt. Rainier’, (x) ‘Nugget’, (xi) ‘Tahoma’, (xii) ‘Triple Perle’, (xiii) ‘Willamette’, or (xiv) ‘Zeus’. The pots were irrigated as required with a drip irrigation system. Pots were fertilized biweekly with all-purpose fertilizer (20:20:20), with a cumulative application of 0.45 g of mineral N supplied to each plant. The plants were grown in a greenhouse for 12 weeks prior to subsequent analysis.

At harvest, plants were carefully uprooted from the soil and assessed for root galling, as described above. The abundance of eggs on each root system was determined by extracting eggs from entire root systems using the sodium hypochlorite technique, as described above. Eggs were collected over a 25-μm sieve, transferred in water into plastic scintillation vials, and store at 4°C for a maximum of 14 days prior counting on an inverted compound microscope.

### Molecular identification of plant-parasitic nematodes

In July 2019, dagger (*Xiphinema* sp.), lesion (*Pratylenchus* sp.), ring (*Mesocriconema* sp.), spiral (*Helicotylenchus* sp.), and stubby-root (*Paratrichodorus* sp.) nematodes extracted from the root zone of hop plants were identified to the species level by sequencing a partial fragment of the 28S ribosomal RNA gene region and the 18S ribosomal RNA gene region. For each genus, one to seven vermiform nematodes were plucked from the July 2019 sample nematode extractions and combined into a single sample for DNA extraction. Total genomic DNA was extracted using the sodium hydroxide nematode digestion protocol (Floyd et al., 2002). The D2-D3 expansion segments located within the 28S ribosomal RNA gene region was amplified using the primers D2A (5′-ACAAGTACCGTGAGGGAAAGTTG-3′) and D3B (5′-TCGGAAGGAACCAGCTACTAGAT-3′). The 18S ribosomal RNA gene region was amplified using the 1813/2646 primer set (Holterman et al., 2006). Amplification reactions contained 2.5 μL of 10X ThermoPol^®^ Buffer (New England Biolabs, Beverly, MA, USA), 0.5 μL of 10 mM dNTPs, 0.5 μL of 25 mM MgCl_2_, 0.5 μL of 10 μM forward and reverse primers, 1.0 μL of nematode digestion solution, and 0.125 μL of *Taq* DNA Polymerase (New England Biolabs, Beverly, MA, USA), brought to a volume of 25 μL. The 28S ribosomal RNA gene region was amplified using the following temperature profile: 5 min at 95°C, 35 cycles (30 sec at 94°C, 45 sec at 55°C, and 1 min at 68°C), followed by a final extension of 5 min at 68°C. The 18S ribosomal RNA gene region was amplified using the following temperature profile: 5 min at 95°C, 5 cycles (30 sec at 94°C, 30 sec at 45°C, and 70 sec at 68°C), 40 cycles (30 sec at 94°C, 30 sec at 54°C, 70 sec at 68°C) followed by a final extension of 5 min at 68°C. All amplification reactions were carried out on a T100 Bio-Rad Thermal Cycler (Bio-Rad, CA, USA), sequenced in both directions by GENEWIZ DNA sequencing services (GENEWIZ Inc., South Plainfield, NJ, USA), and assembled using Geneious Prime v. 2019.2.1 (Biomatters, Auckland, New Zealand). Consensus sequences were compared for similarities within the National Center for Biotechnology Information GenBank database using the Basic Local Alignment Search Tool.

### Statistical analysis

Nematode data from the field and greenhouse experiments were subjected to a one-way ANOVA in SAS Studio (SAS University Edition; version 3.3; SAS Institute Inc., Cary, NC, USA) using the PROC GLM procedure. Differences among treatment means were examined using Tukey’s HSD test (*p* < 0.05).

## Results

### Nematode species recovered from the rooting zone soil of hop cultivars

Plant-parasitic nematode species recovered from the root zone of hop plants grown in a Florida field soil during the three-year trial period included *M. javanica*, *Pratylenchus brachyurus* Filipjev & Schuurmans-Stekhoven, *Paratrichodorus minor* Siddiqi, *Belonolaimus longicaudatus* Rau, *Xiphinema setariae/vulgare* complex Luc, *Mesocriconema xenoplax* Loof & De Grisse, and *Helicotylenchus dihystera* Sher (Table 1). Throughout the study, soil population densities of *P. minor*, *B. longicaudatus*, *X. setariae*/*vulgare* complex, *M. xenoplax*, and *H. dihystera* remained low.

**Table 1.** Molecular identification of plant-parasitic nematode species in the rooting zone of hop cultivars in a Florida field soil.

Common name	GenBank accession	Closest accession	Percent identity (%)	Species
*28S Gene region* ^*a*^				
Dagger	MN922336.1	KX931065.1	97.5	*Xiphinema setariae/vulgare* complex
Lesion	MN922338.1	MG745329.1	98.8	*Pratylenchus brachyurus*
Ring	MN922337.1	FN433872.1	98.1	*Mesocriconema xenoplax*
Spiral	MN922339.1	AB602601.1	99.1	*Helicotylenchus* sp.
Stubby-Root	MN922340.1	MG938546.1	100	*Paratrichodorus minor*
*18S Gene region* ^*b*^				
Lesion	MN911166.1	KY677821.1	98.8	*Pratylenchus brachyurus*
Spiral	MN911167.1	MK796435.1	98.8	*Helicotylenchus dihystera*
Stubby-Root	MN911168.1	KJ934126.1	100	*Paratrichodorus minor*

Notes: ^a^Amplified with D2A/D3B primer set. ^b^Amplified with 1813/2646 primer set.

### Nematode population development in hop yard planting No. 1

Soil population densities of *M. javanica* did not differ significantly among the hop cultivars (Table 2); however, populations were large (> 200 nematodes per 200 mL of soil) in the ‘Chinook’, ‘Centennial’ (WA), ‘Centennial’ (OR), and ‘Cascade’ (OR) cultivars. Population densities of *M. javanica* remained low (< 50 nematodes per 200 mL soil) in the ‘CTZ’, ‘Magnum’, and ‘Triple Perle’ cultivars. Soil population densities of *P. brachyurus* did not differ among the hop cultivars; however, populations were largest in the ‘Magnum’ cultivar (69 nematodes per 200 mL of soil).

**Table 2.** Effect of hop cultivar and planting material source on soil populations of plant-parasitic nematodes and root galling in December 2016 in Hop Yard Planting No. 1.

	Nematodes per 200 mL of soil (No.)		
Cultivar (Source)	*Meloidogyne javanica*	*Pratylenchus brachyurus*	Gall rating (0-10)
‘CTZ’	46	19	1.44 c
‘Cascade’ (OR)	277	42	3.92 abc
‘Cascade’ (WA)	102	26	2.53 bc
‘Centennial’ (OR)	336	23	5.94 ab
‘Centennial’ (WA)	389	27	4.42 abc
‘Chinook’	526	21	6.67 a
‘Fuggle’	127	11	5.83 ab
‘Magnum’	31	69	0.72 c
‘Nugget’	56	29	1.75 c
‘Sorachi Ace’	75	19	3.92 abc
‘Triple Perle’	20	7	1.17 c
‘Willamette’	175	31	3.08 abc
*p*-Value	0.651	0.904	<0.001

**Notes:** Values sharing the same letter within a column do not differ significantly (*p*-value > 0.05), according to Tukey’s HSD.

Root galling varied considerably among the hop cultivars in Hop Yard Planting No. 1. The most severe root galling was observed on ‘Chinook’, ‘Centennial’ (OR), ‘Fuggle’, and ‘Centennial’ (WA). The lowest amount of root galling was observed on ‘Magnum’, ‘Triple Perle’, ‘CTZ’, and ‘Nugget’.

### Nematode population development in hop yard planting No. 2

In December 2017, *M. javanica* population densities in soil did not differ significantly among the hop cultivars; however, populations were largest in the ‘Cascade’ (FL), ‘Centennial’ (WA), ‘Centennial’ (FL), ‘Chinook’ (FL), and ‘Comet’ cultivars (Table 3). In July 2018, soil population densities of *M. javanica* were significantly larger in the ‘Centennial’ (WA) cultivar relative to that of the ‘Cascade’ (FL), ‘Chinook’ (FL), ‘Comet’, ‘Magnum’, ‘Nugget’, and ‘Triple Perle’ cultivars. In December 2018 and July 2019, *M. javanica* populations in soil did not differ significantly among the hop cultivars; however, population densities were consistently large in the ‘Centennial’ (WA), ‘Centennial’ (FL), and ‘Comet’ cultivars.

**Table 3.** Effect of hop cultivar on soil populations of *Meloidogyne javanica* in Hop Yard Planting No. 2.

	*Meloidogyne javanica*/200 mL soil			
Cultivar (Source)	December 2017	July 2018	December 2018	July 2019
‘Cascade’ (WA)	16	68 ab	208	20
‘Cascade’ (FL)	48	3 b	155	29
‘Cashmere’	2	22 ab	89	34
‘Centennial’ (WA)	36	106 a	320	132
‘Centennial’ (FL)	48	69 ab	372	37
‘Chinook’ (WA)	1	17 ab	99	9
‘Chinook’ (FL)	67	0 b	37	5
‘Comet’	27	13 b	278	69
‘Magnum’	1	6 b	234	21
‘Nugget’	5	10 b	85	1
‘Triple Perle’	1	1 b	23	23
‘Zeus’	0	46 ab	33	26
P-value	0.225	0.004	0.922	0.689

**Notes:** Values sharing the same letter within a column do not differ significantly (*p*-value>0.05), according to Tukey’s HSD.

Soil population densities of *P. brachyurus* did not differ significantly among the hop cultivars throughout the first two years of plant establishment in Hop Yard Planting No. 2 (Table 4). Populations of *P. brachyurus* remained numerically low in soil planted with ‘Cascade’ (WA), ‘Cashmere’, ‘Centennial’ (WA), ‘Nugget’, and ‘Triple Perle’.

**Table 4.** Effect of hop cultivar on soil populations of *Pratylenchus brachyurus* in Hop Yard Planting No. 2.

	*Pratylenchus brachyurus*/200 mL soil			
Cultivar (Source)	December 2017	July 2018	December 2018	July 2019
‘Cascade’ (WA)	0	4	23	13
‘Cascade’ (FL)	85	40	24	3
‘Cashmere’	0	13	25	5
‘Centennial’ (WA)	0	3	6	10
‘Centennial’ (FL)	40	22	21	5
‘Chinook’ (WA)	0	33	23	13
‘Chinook’ (FL)	33	7	17	4
‘Comet’	34	8	14	7
‘Magnum’	2	17	98	8
‘Nugget’	0	15	19	3
‘Triple Perle’	1	21	25	7
‘Zeus’	1	71	87	18
*p*-value	0.142	0.085	0.557	0.643

**Notes:** Values sharing the same letter within a column do not differ significantly (*p*-value>0.05), according to Tukey’s HSD.

### Nematode population development in hop yard planting No. 3

Soil population densities of *M. javanica* did not differ significantly among the hop cultivars (Table 5) but in July 2018 and December 2018 soil population densities were consistently large (> 100 *M. javanica* per 200 mL soil) in the ‘Canadian Red Vine’, ‘Mt. Ranier’, ‘Tahoma’, and ‘Willamette’ cultivars. Similarly, soil population densities of *P. brachyurus* did not differ among the hop cultivars throughout the first two years of plant establishment (Table 6) however, population densities were consistently large in the ‘Galena’ and ‘Mt. Rainier’ cultivars.

**Table 5.** Effect of hop cultivar on soil populations of *Meloidogyne javanica* in Hop Yard Planting No. 3.

	*Meloidogyne javanica*/200 mL Soil			
Cultivar	December 2017	July 2018	December 2018	July 2019
‘Canadian Red Vine’	88	128	486	19
‘Galena’	8	146	93	9
‘Mt. Rainier’	31	153	522	5
‘Tahoma’	80	427	339	18
‘Willamette’	12	190	275	10
*p*-value	0.274	0.337	0.309	0.677

**Notes:** Values sharing the same letter within a column do not differ significantly (*p*-value>0.05), according to Tukey’s HSD.

**Table 6.** Effect of hop cultivar on soil populations of *Pratylenchus brachyurus* in Hop Yard Planting No. 3.

	*Pratylenchus brachyurus*/200 mL soil			
Cultivar	December 2017	July 2018	December 2018	July 2019
‘Canadian Red Vine’	1	10	14	8
‘Galena’	11	17	60	18
‘Mt. Rainier’	3	18	63	20
‘Tahoma’	2	4	20	7
‘Willamette’	8	5	28	3
*p*-value	0.559	0.165	0.305	0.217

**Notes:** Values sharing the same letter within a column do not differ significantly (*p*-value>0.05), according to Tukey’s HSD.

### Hop cultivar susceptibility to *M. javanica* in the greenhouse experiment

Root galling differed significantly among the hop cultivars evaluated in the greenhouse experiment (Table 7). The most severe root galling was observed on ‘Comet’, ‘Cashmere’, ‘Canadian Red Vine’, and ‘Chinook’. The lowest amount of root galling was observed on ‘Galena’, ‘Willamette’, and ‘Cascade’. The number of *M. javanica* eggs extracted per plant also differed significantly among the various hop cultivars. The greatest number of eggs was obtained from the ‘Comet’, ‘Cashmere’, ‘Centennial’, ‘Cascade’, and ‘Chinook’ cultivars. The fewest number of eggs was obtained from the ‘Magnum’, ‘Willamette’, ‘Galena’, and ‘Canadian Red Vine’ cultivars.

**Table 7.** Effect of hop cultivar on root galling and the abundance of *Meloidogyne javanica* eggs per plant in the greenhouse experiment.

Cultivar	Gall rating (0-10)	*Meloidogyne javanica* eggs per plant (No.)
‘Canadian Red Vine’	5.33 abc	6,267 c
‘Cascade’	1.83 def	17,253 abc
‘Cashmere’	5.50 ab	29,960 ab
‘Centennial’	3.17 b-e	23,107 abc
‘Chinook’	5.00 abc	16,493 abc
‘Comet’	6.67 a	38,653 a
‘Galena’	0.50 f	4,707 c
‘Magnum’	3.33 bcd	2,453 c
‘Mt. Rainier’	3.33 b-e	10,893 bc
‘Nugget’	3.00 cde	7,307 bc
‘Tahoma’	3.33 b-e	8,880 bc
‘Triple Perle’	3.17 b-e	12,707 bc
‘Willamette’	1.67 def	2,949 c
‘Zeus’	4.67 abc	13,360 bc
*p*-value	<0.001	<0.001

**Notes:** Values sharing the same letter within a column do not differ significantly (*p*-value>0.05), according to Tukey’s HSD.

## Discussion

A diverse range of plant-parasitic nematodes inhabit Florida soils (Porazinska et al., 1999), likely as a result of favorable biotic and abiotic conditions provided by the sandy soils and warm sub-tropical climate characteristic of this growing region. As a result, plant-parasitic nematodes present a significant obstacle to most crops planted in Florida soils. In this study, plant-parasitic nematode species recovered from the root zone of hop planted in a Florida field soil included *M. javanica*, *P. brachyurus*, *P. minor*, *B. longicaudatus*, *X. setariae/vulgare* complex, *M. xenoplax*, and *H. dihystera*. With the exception of *M. javanica* (Brito et al., 2018), this is the first report of *P. brachyurus*, *P. minor*, *B. longicaudatus*, *X. setariae/vulgare* complex, *M. xenoplax*, and *H. dihystera* in the rooting zone soil of hop plants in Florida. For all nematodes, percent identity was greater than 97% for the first five accession numbers in each BLAST search; however, only a single amplicon was sequenced per nematode species, thus restricting the power of this study to identify mixed-species populations within the hop yard. Although these plant-parasitic nematode species were present in the root zone of hop plants, soil population densities remained relatively low for *P. minor*, *B. longicaudatus*, *X. setariae/vulgare* complex, *M. xenoplax*, and *H. dihystera*, suggesting that these particular species are unlikely parasites of major concern in the growing region. The recovery of these nematodes from soil in the rooting zone of the hop cultivars may have been an artifact of the previous natural grass cover in the field prior to establishing the hop yard, or the ryegrass groundcover located between the planting rows, as opposed to direct parasitism of the hop roots. Our findings are in agreement with other studies which have also reported lesion and root-knot nematode parasitizing hop plants in significant numbers in the Pacific Northwest of United States (Maggenti, 1962; Hafez et al., 1992), albeit by more temperate climate nematode species (*P. neglectus*, *P. thornei*, and *M. hapla*) than the nematode species observed in this study.

The abundance of particular plant-parasitic nematode species in the root zone differed considerably among the different hop cultivars planted. Root-knot nematodes are widespread in Florida (Garcia and Rich, 1985), and likely pose one of the most significant obstacles to successful production of hop in the growing region. All hop cultivars that were evaluated were hosts to *M. javanica* to some extent; however, root galling index ratings, egg production, and soil population densities of *M. javanica* were consistently greater on the ‘Canadian Red Vine’, ‘Centennial’, ‘Chinook’, and ‘Comet’ hop cultivars. Root-knot nematode population development was lowest on the ‘Galena’ and ‘Triple Perle’ cultivars. In China, differential host susceptibility of hop cultivars to *M. incognita* has been observed, where the hop cultivar ‘Comet’ also supported substantial root-knot nematode population development, whereas nematode population development was restricted on a locally bred cultivar (Zhang and Zheng-Li, 1989).

No consistent differences in lesion nematode (*P. brachyurus*) soil population densities were observed among the hop cultivars, and destructive sampling to quantify root populations was not performed in order to maintain root health in the newly established hop yard. This makes it difficult to draw definitive conclusions about the host status of *P. brachyurus* on cultivars used in this study. Conducting additional controlled cultivar screening greenhouse experiments, similar to the greenhouse experiment performed with *M. javanica* in this study, could help further delineate the host status of hop cultivars to this nematode. Selecting cultivars that are resistant or tolerant to nematode feeding will be vital to establishing a successful hop industry in Florida.

Plant-parasitic nematodes present an obstacle to any crop grown in Florida and finding suitable hop cultivars that can thrive in the sub-tropical climate as well as tolerate or resist nematode feeding will be vital to the establishment of a sustainable hop industry in the region. In a recently conducted field trial in Central Florida, the cultivars ‘Columbus’ and ‘Chinook’ showed the greatest bine lengths and yield, followed by ‘Neo1’ and ‘Amalia’ (Pearson et al., 2016). In addition to finding vigorous growing hop cultivars, developing effective pest management strategies will also be key to sustaining the hop industry. As a herbaceous perennial crop, opportunities for soilborne pest management are limited on hop. In this study, pre-plant fumigation only provided temporary nematode relief at the time of planting and did not prevent subsequent nematode population development later on in the growing season, as has been observed in other studies on perennial crops (Watson et al., 2017). Nematicides may be able to provide effective management options for use by growers (e.g. fluopyram), alongside other management tools, such as the development of tolerant/resistant hop cultivars through hop breeding programs and adopting management practices that promote soil health and disease suppressive soils.

Overall, our study provides the first report of the plant-parasitic nematode species recovered from soil in the rooting zone of hop in Florida and provides a basis for selecting hop cultivars that restrict plant-parasitic nematode population development in Florida field soils.
